# Not *BCL2* mutation but dominant mutation conversation contributed to acquired venetoclax resistance in acute myeloid leukemia

**DOI:** 10.1186/s40364-021-00288-7

**Published:** 2021-05-01

**Authors:** Xiang Zhang, Jiejing Qian, Huafeng Wang, Yungui Wang, Yi Zhang, Pengxu Qian, Yinjun Lou, Jie Jin, Honghu Zhu

**Affiliations:** 1 Department of Hematology, The First Affiliated Hospital, Zhejiang University School of Medicine, #79 Qingchun Rd, Hangzhou, 310003 Zhejiang China; 2 Key Laboratory of Hematologic Malignancies, Diagnosis and Treatment, Hangzhou, Zhejiang China; 3 Zhejiang University Cancer Center, Zhejiang, Hangzhou China; 4 Institute of Hematology, Zhejiang University, Hangzhou, Zhejiang People’s Republic of China; 5 Zhejiang Laboratory for Systems & Precision Medicine, Zhejiang University Medical Center, 1369 West Wenyi Road, Hangzhou, 311121 China

**Keywords:** Venetoclax, Acquired resistance, Acute myeloid leukemia

## Abstract

Venetoclax (VEN) plus azacitidine has become the first-line therapy for elderly patients with acute myeloid leukemia (AML), and has a complete remission (CR) plus CR with incomplete recovery of hemogram rate of ≥70%. However, the 3-year survival rate of these patients is < 40% due to relapse caused by acquired VEN resistance, and this remains the greatest obstacle for the maintenance of long-term remission in VEN-sensitive patients. The underlying mechanism of acquired VEN resistance in AML remains largely unknown. Therefore, in the current study, nine AML patients with acquired VEN resistance were retrospectively analyzed. Our results showed that the known VEN resistance-associated *BCL2* mutation was not present in our cohort, indicating that, in contrast to chronic lymphocytic leukemia, this *BCL2* mutation is dispensable for acquired VEN resistance in AML. Instead, we found that reconstructed existing mutations, especially dominant mutation conversion (e.g., expanded *FLT3-ITD*), rather than newly emerged mutations (e.g., *TP53* mutation), mainly contributed to VEN resistance in AML. According to our results, the combination of precise mutational monitoring and advanced interventions with targeted therapy or chemotherapy are potential strategies to prevent and even overcome acquired VEN resistance in AML.

**Supplementary Information:**

The online version contains supplementary material available at 10.1186/s40364-021-00288-7.

**To the Editor**

VEN + AZA has become the first-line therapy for elderly patients with AML, and CR + CRi rates of ≥70% have been achieved [1, 2]. Despite this, the 3-year survival rate of patients who receive VEN + AZA is < 40%, mainly due to acquired VEN-R [3]. However, the underlying mechanisms of VEN-R and the status of *BCL2*^*Mut*^ in AML, remain largely unknown [4–6].

To address this question, we retrospectively analyzed nine elderly AML patients with acquired VEN-R at our center from July 1, 2018 until June 30, 2020 (Table 1). *BCL2*^*Mut*^ was detected by PCR combined with Sanger sequencing at VEN-I and VEN-R, but no VEN-R-associated *BCL2*^*Mut*^ was identified (Fig. 1a) [6–9]. Due to the relatively low resolution of Sanger sequencing, these samples were then submitted to TES (Novaseq platform, Illumina), in which 236 recurrently mutated genes in hematological malignancies were included. The average raw sequencing depth on target per sample was ≥1000, and a VAF ≥1% was considered significant. As VEN-R-associated *BCL2*^*Mut*^ was consistently negative, *BCL2*^*Mut*^ was considered dispensable for acquired VEN-R in AML.

**Table 1:** Basic characteristics of patients with acquired VEN-R AML in our cohort

Characteristics	Value
Patients (N)	9
Male/Female (N)	5/4
Age (year)	73 (68–78)
De novo/Secondary (N)	8/1
FAB: M0/M1/M4/M5 (N)	2/1/3/3
Karyotype: normal/abnormal (N)	4/5
Bone marrow blast at venetoclax initiation (%)	62 (23–92)
Molecular feature at venetoclax initiation (N)	
*AML1-ETO*	1
*NPM1* mutation	3
*FLT3-ITD*	4
*DNMT3A* mutation	4
*TP53* mutation	1
*ASXL1* mutation	2
*RUNX1* mutation	2
Bone marrow blast at venetoclax resistance (%)	10.5 (6–74)
Molecular feature at venetoclax resistance (N)	
*AML1-ETO*	1
*NPM1* mutation	2
*FLT3-ITD*	3
*DNMT3A* mutation	3
*TP53* mutation	2
*ASXL1* mutation	2
*RUNX1* mutation	1
Cycles from venetoclax initiation to resistance (N)	3 (3–15)

**Fig. 1:**
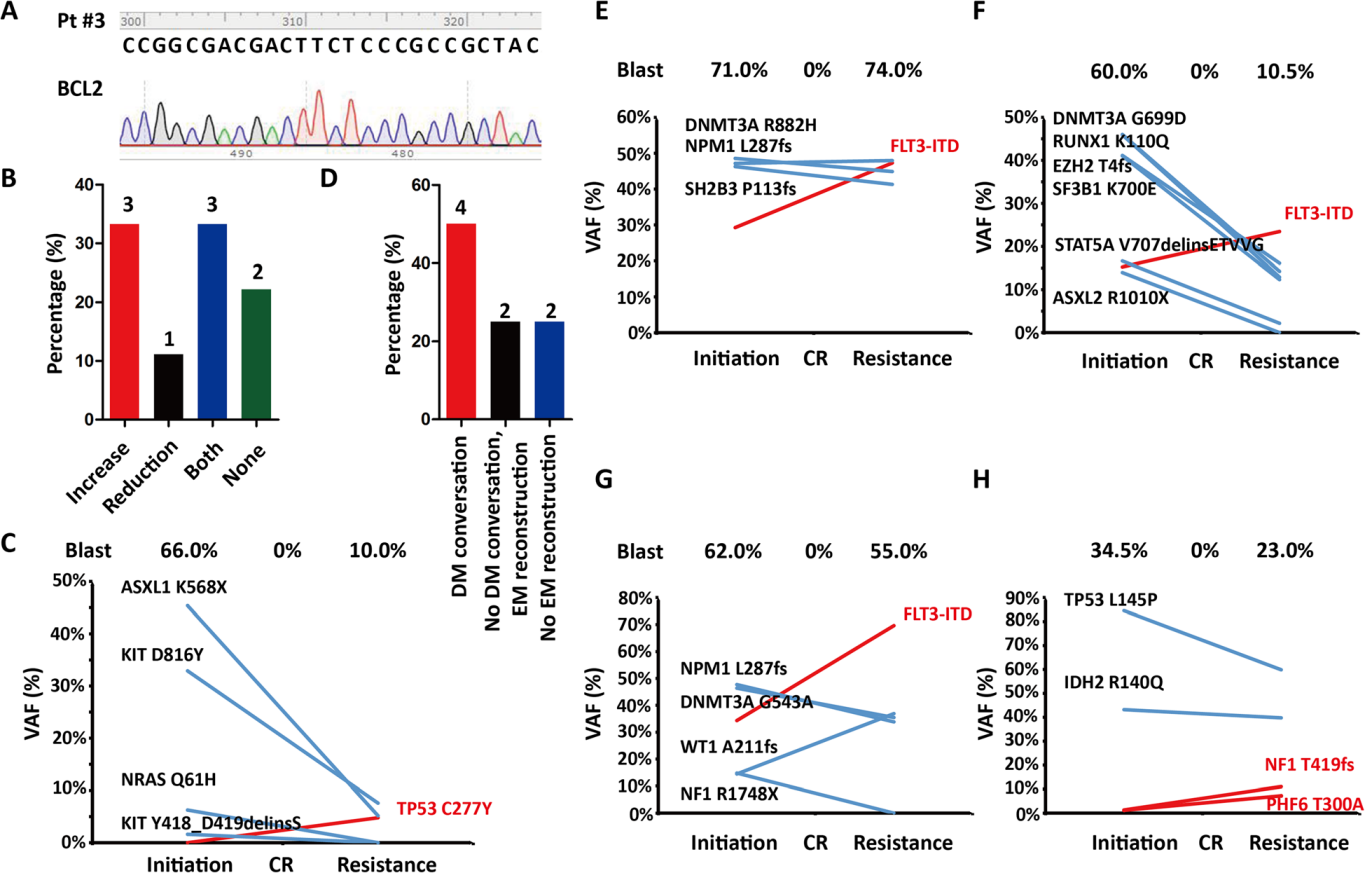
Mechanism of acquired VEN-R in AML. **a** No *BCL2*^*Mut*^ was found in patients with acquired VEN-R AML. **b** Changes in the types of mutational genes in our AML cohort according to VEN-R. **c** The acquired *TP53* mutation played a dominant role in the relapse of Pt #8. **d** Reconstructed existing mutations (EM), especially conversed dominant mutation (DM), were important in acquired VEN-R. **e-g** Expanded *FLT3-ITD*-mediated acquired VEN-R in Pt #3 (**e**), #6 (**f**), and #7 (**g**). **h** The proportion of reconstructed existing mutations in Pt #1

Regarding the difference in the mutational landscape between VEN-I and VEN-R (Supplementary Table 1), the spectrum was skewed in 7/9 patients: 3/7 exhibited a reduction in mutated genes, 1/7 exhibited an increase, and 3/7 showed a reduction in some mutated genes and an increase in others (Fig. 1b). As *TP53* mutation has been demonstrated to confer AML VEN-R [10], newly emerged *TP53* mutation definitely contributed to VEN-R as shown in Pt #8 (Fig. 1c). However, newly emerged mutations in the remaining three patients had relatively low VAFs compared to the dominant mutations, which indicated that these mutations existed in sub-clones and played a minor role in acquired VEN-R.

We next addressed the proportion of reconstructed existing mutations. Excluding Pt #9 without the molecular relapse, 6/8 patients exhibited reconstructed existing mutations, and 4/8 patients showed dominant mutational conversion (Fig. 1d). *FLT3-ITD* is the most common mutation in AML [11], but whether it affects VEN sensitivity remains controversial [1]. In Pt #3, #6, and #7, the VAF of *FLT3-ITD* increased, and it had ranged from a minor mutation at VEN-I to the most common mutation at VEN-R (Fig. 1e–g). Although *FLT3-ITD* was totally absent from Pt #5, *FLT3-ITD* still conferred VEN-R for AML in Pt #3, Pt #6, and Pt #7. In Pt #1, *IDH2*^*R140Q*^ and *TP53*^*L145P*^ mutations were the dominant mutations across the entire treatment course; however, their VAFs decreased, while those of *NF1*^*T419fs*^ and *PHF6*^*T300A*^ mutations gradually increased with AML progression. These findings indicate that minor mutations can expand and possibly contribute to VEN-R (Fig. 1h).

Although VEN-associated *BCL2*^*Mut*^ has been identified in CLL, it was not detected in our AML cohort. There are several possible explanations. First, there was short duration exposure to VEN in AML (AML vs. CLL [months], 5 [3-9] vs. 36[6.5–73]) [12]; second, combination therapy with AZA in AML may have eradicated the emerged *BCL2*^*Mut*^ at an early stage; and third, the standard dose of VEN (400 mg/qd) used in AML patients was not reached in 27% of CLL patients. Theoretically, *BCL2*^*Mut*^ may have mediated VEN-R in patients with AML as the duration of exposure increased, but in reality, combination therapy at a standard dose made the possibility of emerged *BCL2*^Mut^ much lower than in CLL. *BCL2*^*Mut*^ was still negative in our two cases with ≥1-year exposure duration. In contrast to *BCL2*^Mut^, we found that clonal evolution, including newly emerged mutations and reconstructed existing mutations, mainly contributed to VEN-R in AML. For example, newly emerged *TP53* mutation or expanded *FLT3-ITD* could mediate acquired VEN-R in AML, which was also reported by DiNardo [4]. Interestingly, acquired *TP53* mutation also mediated VEN-R in CLL independent of *BCL2*^*Mut*^, and it was more common than in AML. Furthermore, reconstructed existing mutations, especially dominant mutation conversion, appear to be more important than newly emerged mutations in acquired VEN-R. More aggressive clinical strategies are required to overcome this mechanism in acquired VEN-R in AML. In our cohort, three patients with AML with expanded *FLT3-ITD*-mediated acquired VEN-R possibly benefited from dynamic monitoring of *FLT3-ITD* and early addition of an FLT3 inhibitor to prolong the response to VEN. Therefore, the combination of precise mutational monitoring and advanced interventions with targeted therapy or chemotherapy is key to preventing and overcoming acquired VEN-R in AML.

## Supplementary Information

**Additional file 1: Table S1.** Differences in the mutational landscape between patients with VEN-I and VEN-R AML.

## Data Availability

All data generated or analyzed during this study are included in this published article.

## Abbreviations

AML: Acute myeloid leukemia
AZA: Azacitidine
*BCL2*^*Mut*^: *BCL2* mutation
CLL: Chronic lymphocytic leukemia
CR: Complete remission
CRi: Complete remission with incomplete recovery of hemogram
PCR: Polymerase chain reaction
Pt: Patient
TES: Targeted-exome-sequencing
VAF: Variant allele frequency
VEN: Venetoclax
VEN-I: Venetoclax initiation
VEN-R: Venetoclax resistance
